# Circus arts shine a spotlight on antimicrobial resistance in Cambodia

**DOI:** 10.1093/inthealth/ihaf160

**Published:** 2026-01-07

**Authors:** Vanna Moul, Rashaad Wijntuin, Thyl Miliya, Phallina Thong, Phal Chanpheakdey, Sambou Bran, Abhjit Mishra, Aaryan Dahal, Florine van Driessen, Marco Liverani, Tibor Almasy, Hem Vattanak, Neil Murphy, Elke Wynberg, Rusheng Chew, Mom Ean, Osman Khawaja, Chan Davoeung, Voeurng Bunreth, Sarath Kros, Phaik Yeong Cheah, Bipin Adhikari, Thomas J Peto, Sidonn Krang, Paul Turner, Dysoley Lek

**Affiliations:** Action for Health Development, 91 Street 614, Rumchek 2, Sangkat Ratanak, Battambang 2030302, Cambodia; Vrije Universiteit, De Boelelaan 1105, 1081 HV Amsterdam, the Netherlands; Cambodia Oxford Medical Research Unit, Angkor Hospital for Children, Siem Reap17100212, Cambodia; Phare Ponleu Selpak, Anh Chanh, Sangkat Ochar, Battambang 2030902, Cambodia; Mahidol Oxford Tropical Medicine Research Unit, Mahidol University, 420/6 Ratchawithi Road, Ratchathewi, Bangkok 10400, Thailand; Cambodia Oxford Medical Research Unit, Angkor Hospital for Children, Siem Reap17100212, Cambodia; Mahidol Oxford Tropical Medicine Research Unit, Mahidol University, 420/6 Ratchawithi Road, Ratchathewi, Bangkok 10400, Thailand; Mahidol Oxford Tropical Medicine Research Unit, Mahidol University, 420/6 Ratchawithi Road, Ratchathewi, Bangkok 10400, Thailand; University of Amsterdam, Spui 21, 1012 WX Amsterdam, the Netherlands; Department of Global Health and Development, London School of Hygiene and Tropical Medicine, Keppel Street, London WC1E 7HT, UK; N/A Productions, Hong Kong, China; Mahidol Oxford Tropical Medicine Research Unit, Mahidol University, 420/6 Ratchawithi Road, Ratchathewi, Bangkok 10400, Thailand; The Fringe, Street 2.5, Battambang, Cambodia; Mahidol Oxford Tropical Medicine Research Unit, Mahidol University, 420/6 Ratchawithi Road, Ratchathewi, Bangkok 10400, Thailand; Mahidol Oxford Tropical Medicine Research Unit, Mahidol University, 420/6 Ratchawithi Road, Ratchathewi, Bangkok 10400, Thailand; Centre for Tropical Medicine and Global Health, Nuffield Department of Medicine, University of Oxford, New Richards Building, Roosevelt Drive, Headington Oxford OX3 7LG, UK; Mahidol Oxford Tropical Medicine Research Unit, Mahidol University, 420/6 Ratchawithi Road, Ratchathewi, Bangkok 10400, Thailand; Phare Ponleu Selpak, Anh Chanh, Sangkat Ochar, Battambang 2030902, Cambodia; Provincial Health Department, Battambang Provincial Administration Offices, Battambang, Cambodia; Provincial Health Department, Battambang Provincial Administration Offices, Battambang, Cambodia; Provincial Health Department, Siem Reap Provincial Administration Offices, Siem Reap, Cambodia; Mahidol Oxford Tropical Medicine Research Unit, Mahidol University, 420/6 Ratchawithi Road, Ratchathewi, Bangkok 10400, Thailand; Centre for Tropical Medicine and Global Health, Nuffield Department of Medicine, University of Oxford, New Richards Building, Roosevelt Drive, Headington Oxford OX3 7LG, UK; Mahidol Oxford Tropical Medicine Research Unit, Mahidol University, 420/6 Ratchawithi Road, Ratchathewi, Bangkok 10400, Thailand; Centre for Tropical Medicine and Global Health, Nuffield Department of Medicine, University of Oxford, New Richards Building, Roosevelt Drive, Headington Oxford OX3 7LG, UK; Mahidol Oxford Tropical Medicine Research Unit, Mahidol University, 420/6 Ratchawithi Road, Ratchathewi, Bangkok 10400, Thailand; Centre for Tropical Medicine and Global Health, Nuffield Department of Medicine, University of Oxford, New Richards Building, Roosevelt Drive, Headington Oxford OX3 7LG, UK; Communicable Disease Control Department, Ministry of Health, 289 Samdech Pen Boulevard, Phnom Penh, Cambodia; Cambodia Oxford Medical Research Unit, Angkor Hospital for Children, Siem Reap17100212, Cambodia; Centre for Tropical Medicine and Global Health, Nuffield Department of Medicine, University of Oxford, New Richards Building, Roosevelt Drive, Headington Oxford OX3 7LG, UK; National Center for Parasitology, Entomology, and Malaria Control, 477 Betong Street, Phnom Penh, Cambodia; University of Health Sciences, 73 Monivong Blvd, Phnom Penh, Cambodia

**Keywords:** antimicrobial resistance, Cambodia, circus, education

## Abstract

**Background:**

In Cambodia, limited conceptual understanding of antimicrobials and the wide availability of over-the-counter medications increases antimicrobial resistance (AMR) threats. Community-based campaigns are critical to foster fundamental concepts of antimicrobials, their appropriate use and potential consequences of AMR.

**Methods:**

A circus-based drama on AMR was co-designed with a non-profit arts school and local youth groups, in coordination with two provincial health authorities.

**Results:**

Events held across three venues were attended by >1200 people and the accompanying social media campaign received >0.5 million views.

**Conclusions:**

Following the success of the campaign, the circus drama’s key messages are being developed into educational materials for school children nationwide.

## Introduction

Antimicrobial resistance (AMR) is a global health threat, with low- and middle-income countries at increased risk. In Cambodia, AMR rates are high in both the human and animal health sectors.^1,2^ Antibiotic misuse is a major driver of bacterial AMR worldwide. The wide availability of antibiotics over the counter in Cambodia, with poor understanding of antibiotics and their uses, makes it difficult for health authorities to engage with the population on this topic. Performance arts have been shown to successfully communicate key messages on various health topics in Cambodia.^3^ Given the conceptual complexity of AMR tied with the population’s treatment-seeking behaviour, a circus-drama was designed to simplify and communicate the concept of AMR to the population, particularly school children and youth. The main objective of this project was to co-design AMR-circus-drama together with a youth advisory group, circus performers, an interdisciplinary group of experts and the health authorities. This project was connected to current clinical research on AMR in Cambodia: the ACORN (https://acornamr.net/#/) and EDAM (https://www.isrctn.com/ISRCTN15157105) studies. The aim of this project was to harness circus arts, storytelling and social media, to encourage and foster young Cambodians’ awareness of the problem of AMR and its causes. We present the highlights of the process and preliminary reflections from this piloted AMR-circus-drama.

## Methods

The AMR-circus concept was designed by Phare Ponleu Selpak, a Cambodian non-profit arts and circus school, together with a youth group from Siem Pang, who are trained to offer feedback on health-related topics and research.^4^ Content was reviewed by clinical and Ministry of Health (MOH) experts to ensure factual correctness and harmonisation with national messaging. The local youth members were encouraged to prepare educational tools and activities that could highlight AMR concepts through the means of visual arts. The circus’ visual arts school took key messages and produced paintings to display at the events. Given the widespread use of smartphones, QR codes were linked to the project Facebook page, videos and a questionnaire. Videos were shared via social media and through snowballing, to reach wider coverage among the general population. Written communication materials in the form of leaflets, posters and banners were also produced for the audience, and for sharing at local health centres, in alignment with health promotion frameworks and principles.

**Figure 1.**
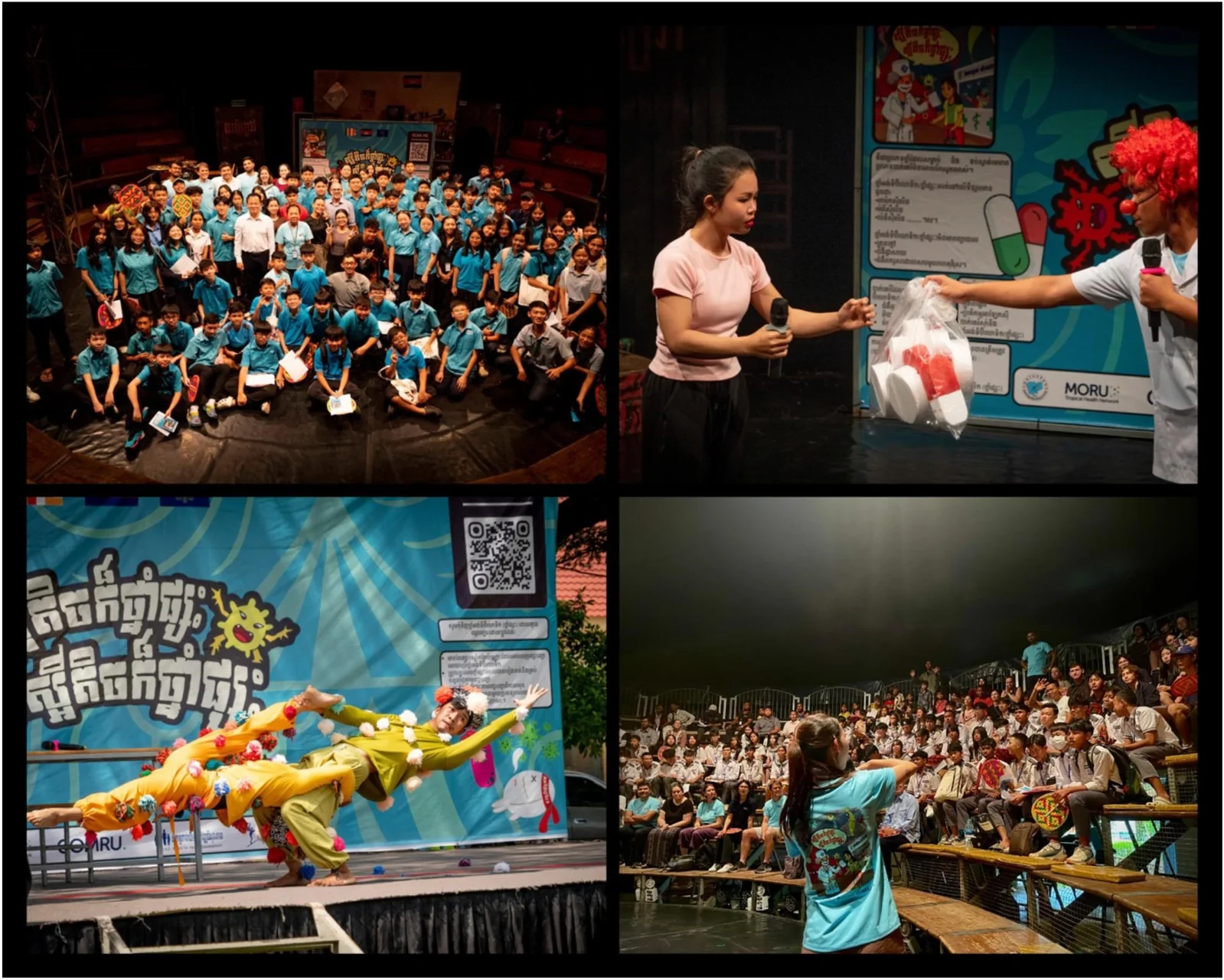
Team photograph with audience from secondary school after first Siem Reap show (top left), clown pharmacist handing giant bag of mixed antibiotics to sick girl in the first Battambang show (top right), antibiotic vs superbug at Roung Chrey High School (bottom left) and audience being invited for questions and answers at a show in Siem Reap (bottom right), May 2025. Photo credit: Nicky Almasy.

Before each performance, a short talk was given by local health authorities. The show consisted of a comic narrative to explain when antibiotics are needed or not. In the beginning, three cast members attend a juggling clown’s drug store, where they are given antibiotics when they do not need them. In the next scene, two acrobats representing an antibiotic and a superbug commence a duel, in which the antibiotic is defeated. Following this, the patients attend a formal health service, where a kind nurse explains for whether there is a need of antibiotics common colds and diarrhoea and potentials of developing resistance. The doctor from the formal health service offers a correct treatment that is without antibiotics. Recovered, the patients regain their strength and go on to perform a series of exciting circus acts and dancing to demonstrate their vigour. Following each performance, to reinforce messages and facilitate interaction with the crowd, a question-and-answer session with prizes for participation was conducted on stage with audience volunteers (Figure 1).

## Results and conclusions

Delivered on 19–20 May 2025 (Battambang) and 22–23 May 2025 (Siem Reap), the circus performances were well received by >1200 audience members. Social media posts have been viewed >500 000 times.

Following the performances, the circus group, project team and key stakeholders met to reflect on the project and discuss the next steps. Four activities were agreed upon:

1. Produce tailored video content that uses relatable youth narratives and visual storytelling to explain AMR in simple, practical terms, especially targeting rural areas where misinformation is high.
2. Pilot a school-based video awareness package that includes short clips, discussion guides and peer facilitation tools, to be adapted by teachers or local health staff.
3. Link these materials to national efforts, for example, AMR Week and existing MOH communication platforms, ensuring that they align with the WHO’s ‘Awareness to Action’ framework and Cambodia’s National Strategic Plan to Combat AMR.
4. Monitor behavioural impact, including knowledge change, attitudes and practices, through youth-led peer interviews or social listening on platforms like Facebook.

Circus performances have rarely been used for health promotion, and yet they can be an excellent way to engage with audiences and imbed important public health messages.^5^ Indeed, theatre has been used for child-friendly AMR engagement (e.g. https://eu-jamrai.eu/childrens-theatre-amr-croatia/#). The co-designing process itself, following the performance, public attendance and widespread appreciation from the local, regional and national authorities, was a successful journey. Further assessments of the impacts from these AMR-circus performances are underway. A qualitative study will evaluate the project and help to inform the development of key messages into materials suitable for dissemination through secondary schools at scale.

## Data Availability

The data underlying this article are available in the article.
